# Particular suicides and psychiatric pathologies: Case Series.

**DOI:** 10.1192/j.eurpsy.2023.2377

**Published:** 2023-07-19

**Authors:** V. Ritorto, M. A. Sacco, S. Gualtieri, P. Ricci, A. P. Tarallo, I. Aquila

**Affiliations:** 1 Institute of Legal Medicine University Magna Graecia of Catanzaro, Catanzaro, Italy

## Abstract

**Introduction:**

Suicide is one of the main cause of death in the world. It’s an important public health problem that is growning on new generations.

**Objectives:**

The manners used to die have always been studied from psychiatry to understand which mental illness induced victim to various ways of suicide. They are multiples and are influenced by different factors. This study includes suicides cases defined “atypical”.

**Methods:**

The cases were examined at the Insitute of legal Medicine Institute of Magna Graecia University of Catanzaro. Methods used were autopsies, and psycological autopsies. The study of the three cases also included the first level toxicological tests..

**Results:**

In the first case, the victim died for cut injuries by using a kitchen knife on the lateral-cervical region of neck; the inspection revealed superficial lesions (test cuts) which, with progressive depth, reached the vascular-nervous bundle. In the second case, in a family contest of apparent welfare, the victim decided to go out home in the middle of the night, to reach an isolated place: there, when he still was in his car, he spilled on his head flammable liquid to accelerate fire effects.

In the case number 3, the victim was found at the bottom of a cliff with earplugs; maybe she was hearing voices in her mind that induced her to death. At home, police found a message on paper about her autopsy will.

**Conclusions:**

The autopsy findings on the cases described are atypical. In every three cases of atypical suicide, the victim was not being treated with therapy, and all victims, probably, were very able to hide their socio-relational malaise. Forensic investigations for the study of suicides must not be limited to the study of fatal injuries. Forensic study about the modalities used to commit suicide wants to be a help to improve knowledge on certain psychiatric pathologies at high risk of suicide.

**Disclosure of Interest:**

None Declared

